# Factors influencing public health financing in British Columbia: A qualitative case study

**DOI:** 10.17269/s41997-025-01125-2

**Published:** 2025-12-11

**Authors:** Mélanie S. S. Seabrook, Mehdi Ammi, Ak’ingabe Guyon, Andrew D. Pinto, Sara Allin

**Affiliations:** 1 North American Observatory on Health Systems and Policies, Toronto, ON Canada; 2 School of Public Policy & Administration, Carleton University, Ottawa, ON Canada; 3 École de santé publique, Université de Montréal, Montreal, QC Canada; 4 Upstream Lab, MAP Centre for Urban Health Solutions, Unity Health Toronto, Toronto, ON Canada; 5 Institute of Health Policy, Management and Evaluation, University of Toronto, Toronto, ON Canada

**Keywords:** Public health, Public health administration, Health policy, Financial management, Budgets, British Columbia, Santé publique, Administration de la santé publique, Politiques de santé, Gestion financière, Budgets, Colombie Britannique

## Abstract

**Objective:**

Though sufficient and stable funds are critical to effective public health systems, existing literature on public health system financing is limited. This study aimed to address this gap by uncovering the salient factors influencing public health system financial decision-making.

**Methods:**

We conducted a qualitative case study of public health system financing in British Columbia, consisting of a jurisdictional review of academic and grey literature, and semi-structured interviews with 14 participants influential in public health budget-setting. Taking an inductive analytical approach, we constructed a conceptual model of the political, structural, and external factors influencing public health funding trends. Building on insight from participants, we identified promising policy considerations for improving the sustainability of public health funding.

**Results:**

Participants identified that external factors such as public health crises and major sociopolitical events create windows of opportunity for investments or cuts. They reported that structurally separating public health budgets from other health service budgets seems to protect public health funding. Political priorities of top decision-makers were highlighted as the most influential political factor, though advocacy has been successful in bringing public health issues onto the political agenda. Strong relationships between public health actors and decision-makers such as senior executives are seen as important for promoting investment in public health programs.

**Conclusion:**

This study sheds light on some of the possible policy strategies for sustaining public health funding, such as inclusion of public health experts in financial decision-making and developing public health performance indicators, which may inform current public health system strengthening efforts.

## Résumé

**Objectif:**

Bien que le financement approprié et stable soit essentiel à l'éfficacité des systèmes de santé publique, la littérature existante sur le financement des systèmes de santé publique est faible. Cette étude visa à combler cette lacune en découvrant les facteurs saillants qui influencent la prise de décision financière en santé publique.

**Méthodes:**

Nous avons mené une étude de cas qualitative centré sur le financement du système de santé publique en Colombie Britannique, comprenant une revue juridictionnelle des littératures académique et grise, et d’entrevues demi-structurées avec 14 participants impliqués dans l’élaboration des budgets de santé publique. En prenant une approche analytique inductive, nous avons construit un modèle conceptuel des facteurs politiques, structurels, et externes qui influencent les tendances de financement en santé publique. Nous avons identifié des possibilités pour améliorer la durabilité du financement de santé publique en nous appuyant sur les suggestions des participants.

**Résultats:**

Les participants ont identifié que les facteurs externes tels que les crises de santé publique et les évènements sociopolitiques créent des fenêtres d’opportunité pour investir ou réduire les budgets. Ils ont signalé qu’une séparation structurelle entre les budgets de santé publique et les autres budgets de services de santé parait protéger les fonds de santé publique. Ils ont aussi souligné que les priorités politiques des décideurs gouvernementaux représentent le facteur politique le plus puissant, mais que la mobilisation d'acteurs en santé publique peut réussir à apporter la santé publique sur l’agenda politique. Les relations solides entre les acteurs en santé publique et les décideurs gouvernementaux sont considérées importantes pour promouvoir l’investissement dans les programmes de santé publique.

**Conclusion:**

Cette étude éclaircit des stratégies politiques pour soutenir le financement stable des systèmes de santé publique, tel qu'inclure des experts en santé publique dans la prise de décision financière et de développer des indicateurs de performance en santé publique, qui pourraient renseigner les efforts actuels visant à renforcer les systèmes de santé publique.

## Introduction

Adequate and stable funding is a critical building block of public health systems, necessary for the effectiveness of long-term prevention work and for appropriate preparation for emerging health threats (Tam, 2021). Public health spending in Canada has been reported to undergo a “boom & bust” cycle of crisis-induced investments followed by budget reductions as the crisis recedes (Graham & Sibbald, 2021; Tam, 2021). Though public health experts have long called for sufficient and stable public health funding (Guyon et al., 2017; Naylor et al., 2003; Tam, 2021), there is limited empirical evidence on how it can be achieved.

Research on public health systems financing is limited to examining public health financing structures in Europe and Canada (Rechel, 2018; Smith et al., 2021), and calculating trends in public health spending (Ammi et al., 2021; Fiset-Laniel et al., 2020). Few have investigated the determinants of public health funding, or priority setting and resource allocation processes for public health systems (Evans, 2021; Jacques et al., 2023; Jolicoeur, 2022).

Political economy theories of health systems financing reforms underscore the need to consider the power of vested interests alongside economic fluctuations in health budget decision-making (Reich, 2019; Sparkes et al., 2019). Studies have empirically tested this theory in Canada, highlighting the influence of political economy factors on public health budget-setting. When deciding how to allocate scarce resources, governments face a trade-off between investing in programs with immediate returns such as acute healthcare versus programs like public health with potentially larger but long-term benefits. Canadian governments tend to prioritize the former (Jacques & Noël, 2022a), where increases in the proportion of the health budget allocated to curative services are associated with decreased allocation to public health, both in the short and long term (Jacques et al., 2023).

Overall, the limited existing evidence suggests that public health budgets in Canada are set based on historical spending and influenced by political cycles and a tendency to prioritize curative care (Jacques et al., 2023). These studies applied political economy theory to uncover quantitatively the factors influencing public health budgets in Canada. Our study builds on this research by using qualitative methods to consider the structural factors related to the budget-setting process alongside political and economic factors influencing public health funding decisions in British Columbia (BC). We aimed to uncover factors with a protective effect over public health budgets and initiators of new investments. Our work contributes to the growing discourse surrounding the strengthening of public health systems by informing future research and policy aiming to safeguard public health funding.

## Methods

We conducted an in-depth longitudinal case study of the public health budget-setting process in BC. We selected BC for this study because it has demonstrated stable public health funding over time relative to other provinces (Ammi et al., 2021), motivating the question of how such trends have been sustained. The first stage of data collection consisted of a jurisdictional review investigating the various stakeholder politics from the Sparkes et al. (2019) political economy framework, including the budget-setting process (*budget politics*) and relevant political shifts, major events, and public health system reforms having an impact on public health financing, building on approaches from other public health jurisdictional reviews (Rechel, 2018; Smith et al., 2021). The second stage consisted of semi-structured interviews with participants from the BC public health system (Appendix 1: Fig. 3) to describe the processes of public health budget-setting and uncover the salient factors influencing decisions. Drawing on political economy theory (Reich, 2019), we applied a deductive and inductive analytical approach to uncover key political and economic factors and elaborate a conceptual model describing their influence on public health funding.

### Jurisdictional review

The jurisdictional review was conducted from Oct. 2022 to Jan. 2023 and consisted of academic and grey literature searches covering 2000–2023. The academic literature search involved keyword searches of the following databases: Scopus, ovid MEDLINE and Healthstar, ProQuest: Canadian Research Index, Pais Index, and Policy File Index. Academic search results were uploaded to Covidence and screened for inclusion. The grey literature search involved targeted hand searching of government sites, public health agencies, non-governmental organizations (NGOs), research institutes, and supplementary Google searches. Canadian newspaper databases were searched by targeting relevant events. Through a process of document content analysis, review data was abstracted and consolidated with interview results. 

### Qualitative interviews

We conducted in-depth semi-structured interviews to access insider knowledge on the processes of public health budget-setting. Recruitment targeted senior executives, program directors, medical health officers, financial officers, policymakers, and elected officials from the provincial government and health authorities, as well as representatives from NGOs. We employed theoretical sampling by drawing on the jurisdictional review to identify potential participants and then using initial interviews to inform further sampling (Coyne, 1997). We also aimed to collect a sample of perspectives that span the categories of stakeholders involved in health financing reform proposed in the political economy framework by Sparkes et al. (2019), relating to *interests, leadership, bureaucracy, budgets, beneficiaries,* and *external actors*. Participants were also asked to recommend other potential participants, leading to a mix of snowball and respondent-driven sampling (Patton, 2014). We conducted interviews between January and May 2023 and ended recruitment when we reached data saturation, meaning no new themes were emerging, and when we had interviewed all types of stakeholder groups to the extent possible (see “Limitations”).

This study was approved by the University of Toronto Research Ethics Board (protocol # 43336), and participants signed a consent form prior to participating in an interview. The interviews were semi-structured with open-ended questions regarding the processes of public health budget-setting and factors influencing decision-making informed by political economy lines of inquiry. We used an interview guide (Appendix 2: Table 5) and tailored it to each participant to capture their unique expertise and context. Interviews lasted between 30 and 60 min, and were conducted and recorded over Zoom. Recordings were transcribed using otter.ai software and revised by the study team for accuracy. Interview quotes were edited for clarity, conciseness, and anonymity. Five participants contributed to member-checking a summary of our synthesized results.

### Data analysis

Drawing on grounded theory, which consists of inductive research to develop a conceptual model based on the data collected (Creswell & Poth, 2018), we took an exploratory approach to understanding the phenomenon of public health financing. In line with grounded theory methods, we revised the literature searches and interview guides to iteratively pursue and capture new and important lines of inquiry (Creswell & Poth, 2018). Interview transcripts were double-coded inductively using Nvivo software, starting with a preliminary codebook derived from the interview guide and research questions. In organizing our results, we grouped factors influencing public health financing decisions and outcomes into three categories — external, structural, and political, deductively informed by political economy theory (Reich, 2019; Sparkes et al., 2019). We then constructed a conceptual model illustrating the influential pathways of identified factors on public health financing outcomes (Creswell & Poth, 2018).

## Results

### Jurisdictional review data

The academic literature search yielded 674 citations, of which 15 met the inclusion criteria. The grey literature search produced 36 sources deemed relevant and included. Review sources can be found in the Supplementary Resource and the PRISMA diagram can be found in Appendix 3: Fig. 4.

### Participant characteristics

Of the 35 individuals invited to participate in an interview, 14 accepted and were interviewed, with past or present positions in public health practice, finance, and leadership across four of the five regional health authorities, the Provincial Health Services Authority (PHSA), the BC Ministry of Health, and NGOs (Table 1).

**Table 1:** Participant characteristics

System level	Ministry of Health	3
	Health Authority	10
	Non-governmental organization	4
Positions	Senior Executives	7
	Executive Directors	3
	Medical Health Officers	3
	Financial Officers	3
	Interest group representatives	5
	Consultant	1
Years worked in the BC health system	< 10	2
	10–19	7
	20 +	5

The sample includes 14 participants, but the table categories do not sum to 14, as many participants have held and spoken about multiple different positions, and there is some overlap in the types of positions (e.g. a Senior Executive could also be a Medical Health Officer or a Financial Officer)

### Budget-setting process

**Fig. 1:**
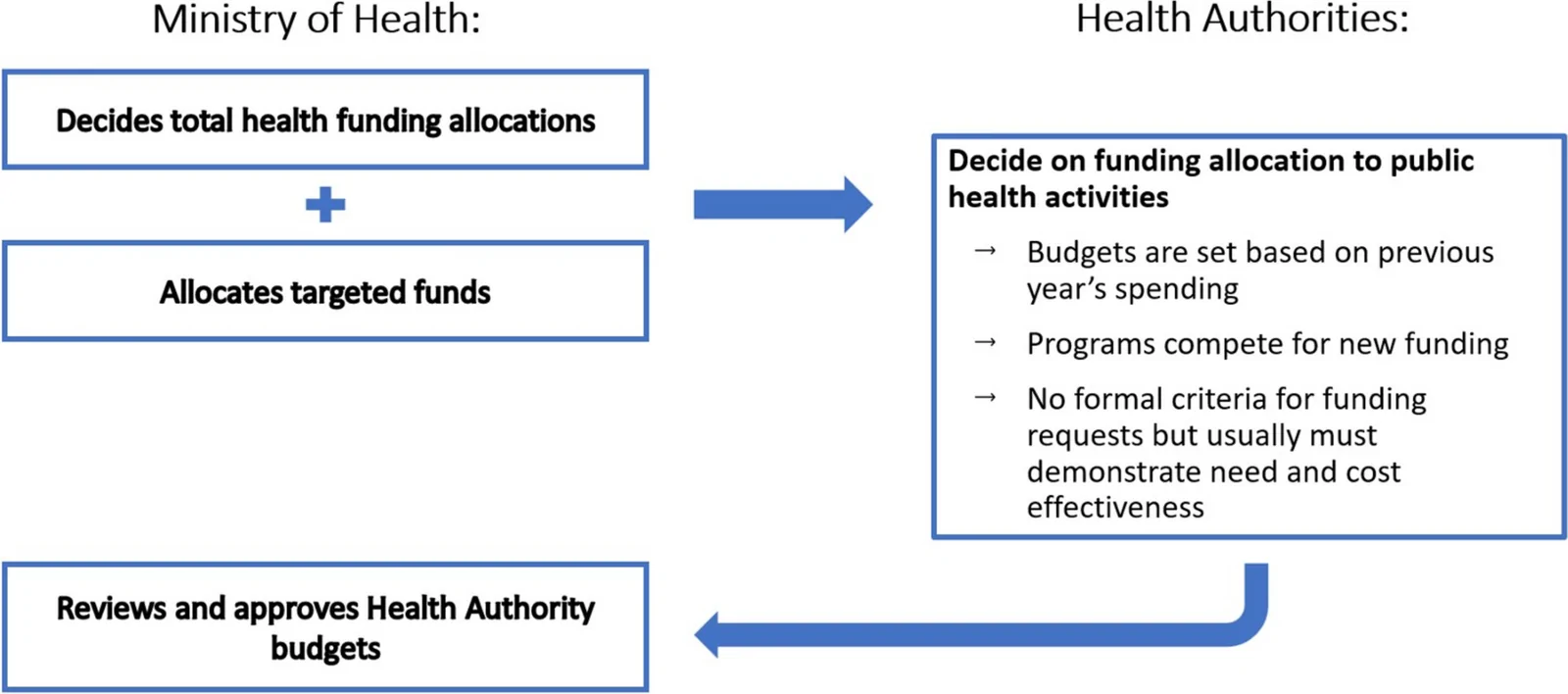
Simplified overview of the budget-setting process for public health services

BC public health programs are primarily funded by the provincial Ministry of Health. The Ministry of Health decides on the total health funds to be allocated to health authorities (approved by the Treasury Board & Legislative Assembly) (Fig. 1). Provincial and regional health authority senior executive teams then allocate funding among their various health services, including “Population Health & Wellness.” Health authority budgets and applications for targeted funds are returned to the Ministry of Health for approval. Any changes to the health authorities’ budgets must undergo multiple levels of approvals, depending on the size of the change, starting with the relevant VP and then the Chief Financial Officer. The full senior executive approves large budget changes, and significant budget changes may require approval from the Ministry. Health authorities report back to the Ministry on the use of targeted funds at the end of the year. The Ministry of Health also allocates funds to partner organizations such as NGOs (e.g. the BC Alliance for Healthy Living) through its Population & Public Health division. Public health policy advisory committees coordinate public health system planning, including preparing funding proposals. A detailed description of BC’s public health budget-setting process can be found in Seabrook (2024).

### Factors influencing public health funding in BC

This section outlines the influential factors in public health system financial decision-making and outcomes, grouped into external, structural, and political factors.

#### External factors

External factors such as major social or economic events and public health emergencies pressure governments to engage and respond through investing in or cutting public health budgets (Table 2). Participants noted that public health crises are one of the most impactful factors influencing public health funding, usually triggering large investments. However, crises tend to concentrate resources on emergency response, redirecting resources from other public health functions.

“The sad thing is that an epidemic of any kind, whether it was SARS, or H1N1, or in this case COVID-19, increases attention on public health. … But it narrows the vision of public health to infectious disease management, and it’s so much more than that.”

Public health crises bring to light vulnerabilities in the system, which have prompted public health system renewal in the past, following the SARS outbreak in 2003 (Ministry of Health Services, 2005), and recently in response to COVID-19 (Ministry of Health, 2024). Public and government officials’ understanding and appreciation for the public health system increase following public health crises, which can lead to support for new investment.

Major sociopolitical events also have an important external influence on public health funding by introducing new fiscal pressures such as during a recession or by creating opportunities. For example, the 2010 Vancouver Olympics opened a window of opportunity for a new public health campaign (ActNow), becoming the most impactful social event leading to investments in public health in the province in the past two decades. The campaign was propelled by a collaboration between public health officials and advocates and facilitated by political buy-in. The Premier’s ambition of being the healthiest place to ever host the Olympics was acted on by the BC Healthy Living Alliance, which conducted an evidence review to produce policy recommendations for improving BC’s population health (Public Health Agency of Canada, 2009). Provincial Health Officer Dr. Perry Kendall and the Assistant Deputy Minister of Population Health and Wellness coordinated with the BC Healthy Living Alliance to present the strategy to the provincial Select Standing Committee on Health, which endorsed it and recommended doubling the public health allocation of the health budget from 3 to 6% (Select Standing Committee on Health, 2004). Deputy Minister of Health Dr. Penny Ballem then made the case for funding the strategy to Cabinet and gained their approval (Public Health Agency of Canada, 2009). The ActNow strategy was launched in 2005, accompanied by investments allocated across NGOs, various ministries, local government, and regional health authorities totalling $98.2 M over 3 years (PHAC, 2009); Foster, 2011).

**Table 2:** External factors impacting public health funding

Date range	External factor	Potential or actual impact on public health funding
2000	Walkerton, Ontario waterborne *Escherichia coli *outbreak	Highlighted the importance of effective public health oversight of water systems, triggering investments in BC water system upgrades totalling $109 million in 2002
2003	SARS outbreak	Revealed limits to epidemiologic investigation processes and health system surge capacity, initiating the renewal of public health systems across Canada. Prompted federal investment in public health of $400 million over 3 years, with $51 million allocated to BC
2004–2010	2010 Olympics hosted in BC	Triggered the development of the ActNow strategy, involving cross-sectoral collaboration and many major investments in public health between 2005 and 2010, including $25 million to the BC Healthy Living Alliance in 2006, $5 million to the Union of BC municipalities in 2005, and $15 million over 3 years for cross-ministry collaboration. $8 million, $16 million, and $24 million was allocated for public health to regional health authorities in the 3 consecutive years leading up to the Olympics
2008–2010	Economic Recession	Austerity pressures led to major cuts in government spending, including a direction from the Minister of Health to cut health authority public health budgets by 10% in 2010
2009	H1N1 pandemic	Provision of 2 million antiviral treatments and 1.8 million vaccinations, though related expenditures were not reported publicly
2016–present	Toxic drug crisis	Commitment of $322 million over 3 years by the BC government to respond to the crisis
2020–2023	COVID-19 pandemic	Led to unrestricted spending on pandemic response

Documentary sources are listed in the Supplementary Resource

#### Structural factors

Structural factors include the organizational and policy features of the health system that shape decision-making (Table 3). Participants reported that public health budgets have been fairly stable since the 2001 reform that created the regional health authorities and set the original public health base budgets. More recently, some regional health authorities such as Vancouver Coastal Health separated public health funds from their sub-region budgets that cover multiple health programs and are managed by sub-regional VPs. The health authority’s consolidated public health budget was placed under the management of the Chief Medical Health Officer and/or a VP of Public Health. Participants observed that this structure helped prevent the reallocation of funds to healthcare activities. They also noted that including Chief Medical Health Officers and VPs of Public Health on health authority senior executive teams allowed public health expertise to inform funding decisions and enabled public health leaders to advocate for new public health funding directly to other decision-makers at the regional level.

“I think those positions are really key to educate and share about the value of public health. … Just having [the Chief Medical Health Officer’s] perspective at the table, it’s been really important. The questions they ask, their thinking, their experience is unique, and I think they’re invaluable. Now I think back and I can’t even imagine why they weren’t here before.”

Participants also reported that the *Public Health Act* assigns special powers to Medical Health Officers related to crisis spending and that though unlegislated, the BC Public Health Guiding Framework can be useful for providing performance measures such as smoking rates and immunization rates to guide resource allocation. Finally, many highlighted challenges with workforce availability in the public health system. Complex approval processes complicate repurposing unused funds from vacancies, leading to public health budget surpluses.

**Table 3:** Structural factors influencing public health funding

Date	Structural factor	Potential or actual impact on public health financing
2000	*Budget Transparency & Accountability Act*	Requires ministries and other government entities to publish yearly service plans and budgets
Dec 2001	Consolidation of the 52 regional health authorities into 5 and establishment of the PHSA under the *Health Authorities Act*	Centralized public health system organization and resources and set the original health authority base budgets
2005	BC Framework for Core Functions in Public Health	Meant to act as a baseline of public health services to be delivered and inform resource allocation. Recommends investments in public health capacity
2008	*Public Health Act*	Lays out responsible actors and their powers to fulfil public health functions, primarily regarding health protection functions
2008–2010	Creation and dissolution of the Ministry of Healthy Living & Sport	Created to manage the implementation of the ActNow strategy and public health functions more broadly. Separated some public health funds from the larger Ministry of Health budget
2013	Establishment of the First Nations Health Authority (FNHA)	Responsibility for the delivery of health services (including public health) for First Nations was transferred from the federal government to the FNHA
2013 (updated in 2017 and 2024)	BC Guiding Framework for Public Health	Provides performance measures for public health programs to help guide resource allocation

Documentary sources are listed in the Supplementary Resource

#### Political factors

Political factors consist of the value judgements that influence budget decisions and the power dynamics involved in stakeholder interactions and relationships (Table 4). Participants reported that public health is rarely on the political agenda, except in crisis times such as epidemics, since public attention tends to focus on curative care system challenges. However, several participants noted that advocacy can play an influential role in bringing public health issues to the attention of senior decision-makers at all levels of the public health system. In budget-setting processes for health, programs compete for new funding, and participants agreed that curative health services are usually prioritized over public health, in part due to the limited public health expertise of most health budget decision-makers. However, public health system leaders work to demonstrate the value of funding public health programs through their relationships with decision-makers. To accomplish this, they make strategic use of evidence in funding proposals, noting that aligning evidence with political priorities and providing economic rationales is most effective in achieving funding approvals.

“I believe that the informal channels—where you have good government relations, warm relationships, and where you are making the case that what you’re trying to advance actually aligns with government frameworks and priorities—that’s where you have the best success in making changes.”

**Table 4:** Political factors influencing public health funding

Political factor	Potential or actual impact on public health funding
Competition with curative healthcare sector	Curative services are often prioritized for funding over public health, and public health funds have sometimes been redirected to address healthcare deficits in the past
Political agendas	Public health seldom reaches the political agendas of elected officials
Public attention	The public tends to pressure the government to address healthcare issues that they interact with directly, such as wait times for curative services
Public health advocacy	Public health advocacy helps bring public health issues to the attention of decision-makers through formal and informal channels
Decision-maker expertise	Decision-maker level of understanding of public health influences their prioritization of public health programs for funding
Relationships between public health actors and decision-makers	Strong relationships between public health actors and decision-makers leads to more informed decision-making around resource allocation to public health programs
Relationships across public health actors	Public health actors collaborate across the Ministry of Health, health authorities, and NGOs to coordinate efforts around funding requests and advocacy
Transparency in decision-making	Transparency around decision-making for public health budget-setting is limited, which leads to distrust and barriers to effective planning
Use and framing of evidence	Framing evidence in alignment with political and economic priorities in funding proposals increases their likelihood of being approved

## Discussion

This study uncovered the salient factors that influence public health financing decisions and outcomes in BC. Over the past two decades, the largest public health investments occurred as the result of the combination of opportunistic public health advocacy during major events and pressure prompted by public health crises. The ActNow campaign was considered effective in large part due to strong collaboration between stakeholders and support from senior decision-makers. This process is facilitated by good relationships between public health leadership and decision-makers, particularly when public health system voices are included at senior decision-making tables.

Participants confirmed that when public health budget cuts and reallocations to other services did occur, they were generally attributed to public health programs being of lower priority relative to more urgent healthcare financial needs, as reported in other studies (Jacques et al., 2023). Competition with curative healthcare for funding is also considered one of the main barriers to new funding for public health; likewise, public health budgets in European countries were found to be more vulnerable to austerity cuts than acute care budgets (Rechel, 2019). Public health leaders employ different framings to sway decision-makers, and find that presenting evidence on return-on-investment, prevention of future healthcare pressures, and alignment with government priorities are most effective in achieving investment in public health.

**Fig. 2:**
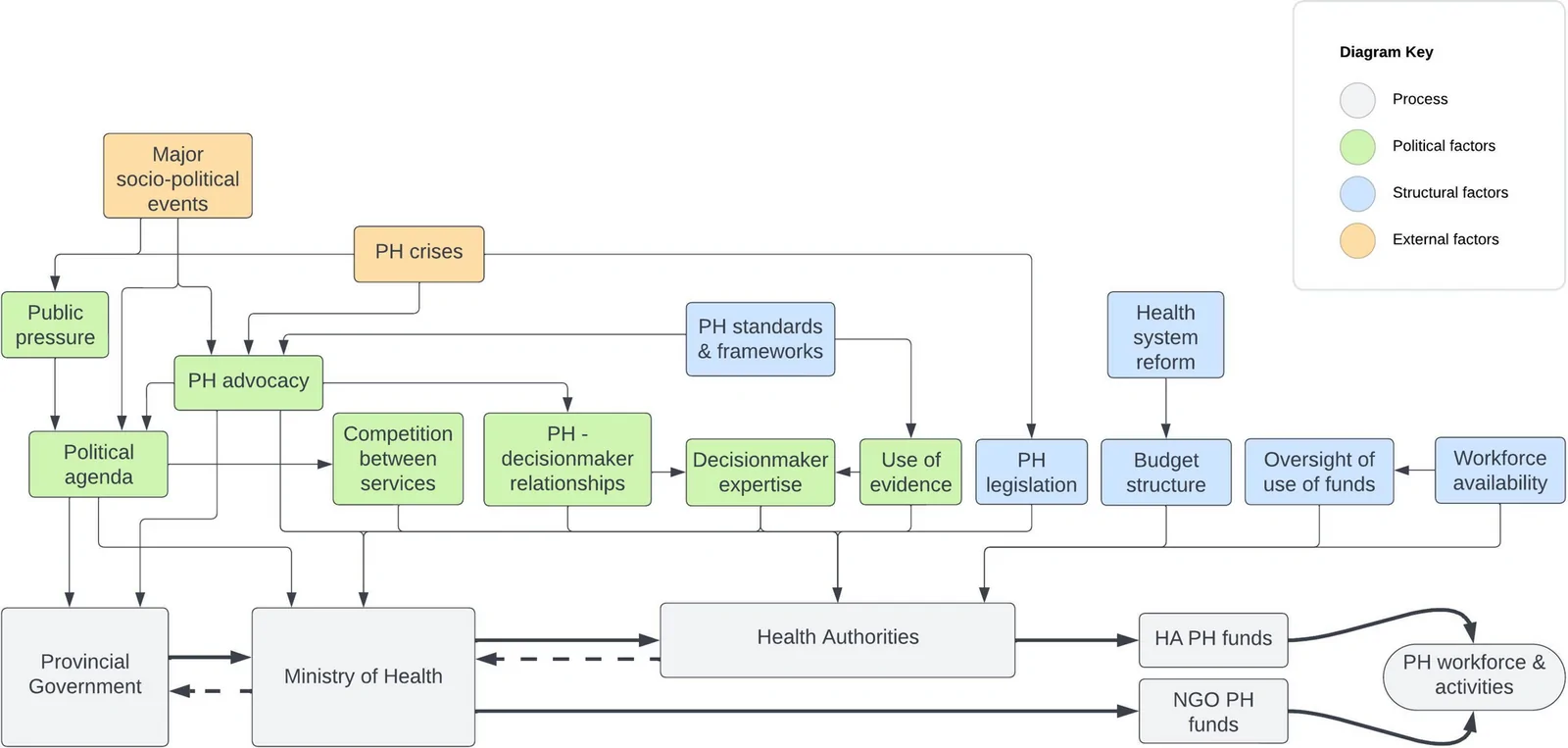
A conceptual model of the factors influencing public health funding. Abbreviations: PH, public health; HA, health authorities; NGO, non-governmental organizations

From this case study and building on political economy theory and the current public health financing literature, we formed a conceptual model of factors influencing public health funding. The model (Fig. 2) portrays how the three layers of influential factors described in the results interact with each other and the budget-setting process. This model allows us to conceptualize the system of influences on public health funding, and begin to identify pathways that could be moderated by policy and practice strategies to strengthen public health financing. It is important to note that the model does not represent all potential relationships between factors, but rather the most salient ones highlighted in the BC case. Though the model is limited to describing the case of BC between 2000 and 2023, we attempt to generalize the factor concepts and relationships in the following discussion so that they may hypothesize how these processes may occur in future or in other jurisdictions.

### Process (in grey)

The process portrayed in this model is based on the BC public health budget-setting process, starting with provincial Ministry of Health budget-setting, followed by resource allocation to the health authorities and NGOs through the Ministry’s Population & Public Health division, and finally health authority resource allocation to public health activities. Though this process is mainly top-down, the dotted arrows refer to the ways in which each level may submit budget proposals to senior decision-makers (i.e. Ministry of Health treasury board submissions, health authority business cases).

### External factors (in orange)

Two examples of major sociopolitical events that occurred in the BC case study were the 2008 recession and the 2010 Olympics. These two contrasting events demonstrate how major events either constrain government finances leading to cuts, or create opportunities to bring public health onto the political agenda. Major events tend to have a strong influence over political processes due to public engagement and interest, fostering political will. They may also be taken up by politicians and advocates to advance their goals, as was evident in the ActNow campaign motivated by the upcoming Olympics. This aligns with political economy theory, which underscores the importance of vocal stakeholders in determining reform trajectories (e.g. public health advocates) (Sparkes et al., 2019). It also highlights the facilitating role played by the Provincial Health Officer in advancing public health policy as “Everybody’s Expert” (Cassola et al., 2022), though further comparative analysis is required to determine how Chief Medical Officer of Health model types impact public health funding in the long run. Major sociopolitical events will likely continue to offer opportunities for public health actors to introduce public health issues onto the political agenda, though strategic framing is needed to align public health goals with the politics of the event.

Though widespread public health crises tend to trigger public pressure for government action, some crises are concentrated in specific populations, such as the toxic drug crisis in BC. These tend to garner less public attention, and thus depend on interest group pressure, such as public health advocacy, to push for government action. Public health legislation may give special powers to public health leadership in crises, such as the power to fund a response, as BC’s *Public Health Act* allows. Funding triggered by public health crises may follow a “boom & bust” cycle (Graham & Sibbald, 2021), but does not always (H1N1 being one such exception), and tends to concentrate in emergency response spending, crowding out other public health functions, such as promotion (Ammi et al., 2024). Nonetheless, they increase decision-maker awareness of public health system functions and importance, which can lead to interest in public health system strengthening, as occurred across Canada post-SARS and in recent years of the COVID-19 pandemic (Jolicoeur, 2022; Naylor et al., 2003).

### Structural factors (in blue)

Health system reforms change the structure of the public health system, impacting who manages public health programs, how resources are allocated to them, and how budgets are structured. Based on BC public health spending trends, we can predict that base budgets set by a system reform will stay largely stable unless impacted by a major sociopolitical event or public health crisis. Though public health legislation and frameworks can guide financial decision-making by providing rationale for funding, their influence is limited in BC as funding mandates are not legislated.

Budget structure refers to whether public health funds are consolidated into a distinct “public health” budget or integrated with other service budgets, and whether they are managed by public health or non-public health directors. In BC, health authorities that transitioned to a consolidated public health budget under public health management were less likely to experience reallocation of funds to other non-public health services. In Quebec, public health budgets are integrated with other health and social services, and public health funds are reportedly commonly redirected to other services despite legislation banning this (Labadie, 2020). Therefore, our results lend support to maintaining some boundaries between public health and acute care versus integrating them (Trein, 2017), particularly in the context of rising healthcare costs.

The oversight of the use of funds impacts how health funds are spent across services. The BC case study demonstrated that complex oversight measures seemed to prevent public health from repurposing and spending allocated funds. However, oversight that aims to protect public health funds specifically, such as ring-fenced targeted funds, seems to be effective in ensuring funds are spent on public health.

Workforce availability refers to the ability of public health divisions to hire and retain public health workers. As workforce pay accounts for most of public health spending, hiring challenges have a large impact on spending levels. This is further limited by barriers to repurposing funds posed by complex approval processes. This dependent relationship between public health workforce and financing reflects the health system building blocks framework,^1^ which describes the interrelatedness of the core components of all health systems (World Health Organization, 2010).

### Political factors (in green)

The competition between health services for new resources and the tendency to prioritize curative services can be in part explained by the “Rule of Rescue” or “identifiable victim effect” (Cookson et al., 2008), which suggests that humans are more likely to allocate resources to an identifiable victim in crisis than to spend resources preventatively even with a higher cost–benefit. This broadly predicts, as we found in BC, that decision-makers are likely to prioritize allocating resources to healthcare issues they deem “urgent” over investing in long-term public health prevention.

The balance between spending on curative healthcare and public health is highly linked to the political agenda of elected officials. Described as a quiet policy due to its relatively small budget allocation with impacts mainly seen in the long term, public health rarely reaches the political agenda (Jacques & Noël, 2022a). Further, it has no strong constituency pressuring the government on its behalf (Jacques & Noël, 2022b). In contrast, curative healthcare represents a “loud” policy, with a large budget providing income for diverse health professions, and institutions representing strong interests (Jacques & Noël, 2022b). A study interviewing elected officials in Quebec found healthcare lobby power was more powerful than that of public health (Jolicoeur, 2022), reflecting the strong influence of *interest group politics* discussed in the political economy of health financing reform (Sparkes et al., 2019). Such political influences seem to overwhelm the expected coordination of public health and healthcare sectors towards common goals by state-governed health systems (Trein, 2017). Curative healthcare also provides direct short-term impacts evident to the public (Jacques & Noël, 2022b), leading to public pressure on decision-makers to prioritize healthcare, in contrast with public health which garners little public attention. The invisibility of public health has been reported across countries including the United Kingdom (Evans, 2021).

Despite limited lobbying power, public health advocacy can bring public health issues onto the political agenda through strategic alignment with political priorities and relationships with decision-makers. Advocacy efforts can sometimes lead to contracted partnerships between NGOs and public health authorities, as happened with the ActNow campaign and other regional public health issues in BC (Public Health Agency of Canada, 2009).

In times of austerity such as the 2008 recession, decision-makers may frame public health as an administrative service to lower the risk of backlash for budget cuts, as the public is more likely to accept cuts to what they perceive as non-essential services (Jolicoeur, 2022), a strategy part of *leadership politics* (Sparkes et al., 2019). This tactic has been seen in other jurisdictions; for instance, the Quebec government implemented a 33% cut to regional public health budgets by framing them as administrative inefficiencies (Fiset-Laniel et al., 2020).

Relationships between public health actors and decision-makers were also reported as highly influential in determining whether decision-makers pay attention to, and as a result, fund public health programs, reflecting key influences of *bureaucratic politics* theorized by Sparkes et al. (2019). These relationships increase decision-maker awareness of public health issues and help align public health goals with decision-maker priorities. Strategic use of evidence can also sway the prioritization of health services by increasing decision-maker awareness of public health needs, impacts, and cost-effectiveness. However, Evans’ model of public health funding in the United Kingdom predicts that politician ideology ultimately trumps public health evidence (Evans, 2021).

## Policy considerations

While the implications we can draw from a single case study are limited, we present policy considerations for protecting and promoting stable public health funding in BC and lessons to inform change in BC and other jurisdictions.

Including public health leaders in financial decision-making such as budget-setting seems to have a protective effect on public health budgets and has been recommended by others, notably Strumpf (2020). Relatedly, consolidating public health budgets under public health management at the regional level seems to prevent the reallocation of public health funds to other services, and has also been suggested by Denis et al. (2020).

This case study demonstrated how partnerships between external public health organizations and the health system led to more effective advocacy for new and long-lasting investments in public health. Such partnerships could be sustained through ongoing financing of public health NGOs and community groups as well as clear channels for consultation and collaboration in programming.

Participants called for greater transparency in decision-making and clarity in financial processes, both publicly and within health system leadership, which they believe would help create a more equitable allocation process and improve long-term planning.

Participants voiced the importance of basing funding requests in evidence of population needs and program effectiveness and called for dedicated resources for health surveillance, which could inform health system-wide decision-making. Relatedly, they pointed to the need for developing a standard performance indicator reporting system to help demonstrate the impact of public health programs to decision-makers and identify areas requiring investment.

Technical changes may help overcome barriers to spending allocated public health funds and increase the predictability of public health financing. For instance, participants support a shift from targeted funds to base budget increases to allow for sustainable program implementation, also reported in Dedewanou et al. (2023). Reviewing approval processes for repurposing funds may also better enable allocated funds to be spent on public health programs.

## Limitations

Our recruitment of a diverse participant sample was limited by the availability of public contact information and our networks. We faced non-response from invited “elite” participants including elected officials and senior decision-makers, limiting our ability to investigate *leadership politics* and the key factors influencing final budget decision-making*,* though this line of inquiry has been explored elsewhere (Jolicoeur, 2022). We elected not to interview *beneficiary politics* stakeholders as it would require different, community-based methods, and *external politics* stakeholders as they did not prominently emerge in the jurisdictional review or interviews. We encourage future research to investigate public perspectives on public health financing more directly, building on Jacques et al. (2025), and the influence of external actors such as the media and lobbyists (Jolicoeur, 2022; Sparkes et al., 2019).

**Fig. 3:**
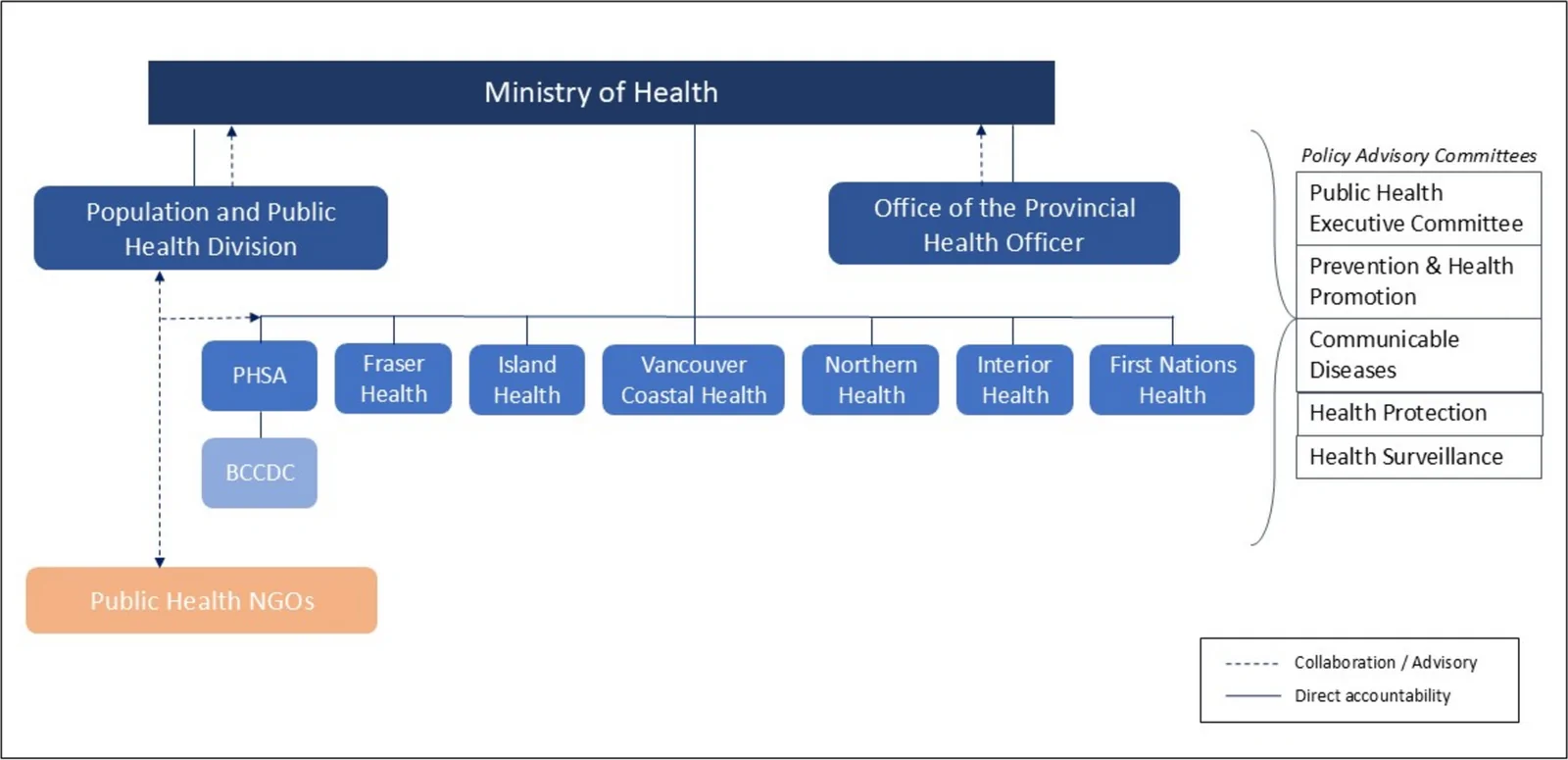
Structure of BC’s Public Health System (adapted from Seabrook, 2024), depicting the organization and relationships between actors involved in public health financing in BC. The Ministry of Health is responsible for the strategic direction of the public health system and allocates funding for all health programs, including public health, to the various health authorities. The regional health authorities, the Provincial Health Services Authority housing the BC Centre for Disease Control, and the First Nations Health Authority are responsible for delivering public health services. Various public health NGOs, such as the BC Alliance for Healthy Living, also receive funding from the Ministry to organize public health programs. Public health policy advisory committees coordinate public health system planning, including preparing funding proposals. Abbreviations: PHSA (Provincial Health Services Authority); BCCDC (British Columbia Centre for Disease Control); NGO (Non-Governmental Organization)

**Fig. 4:**
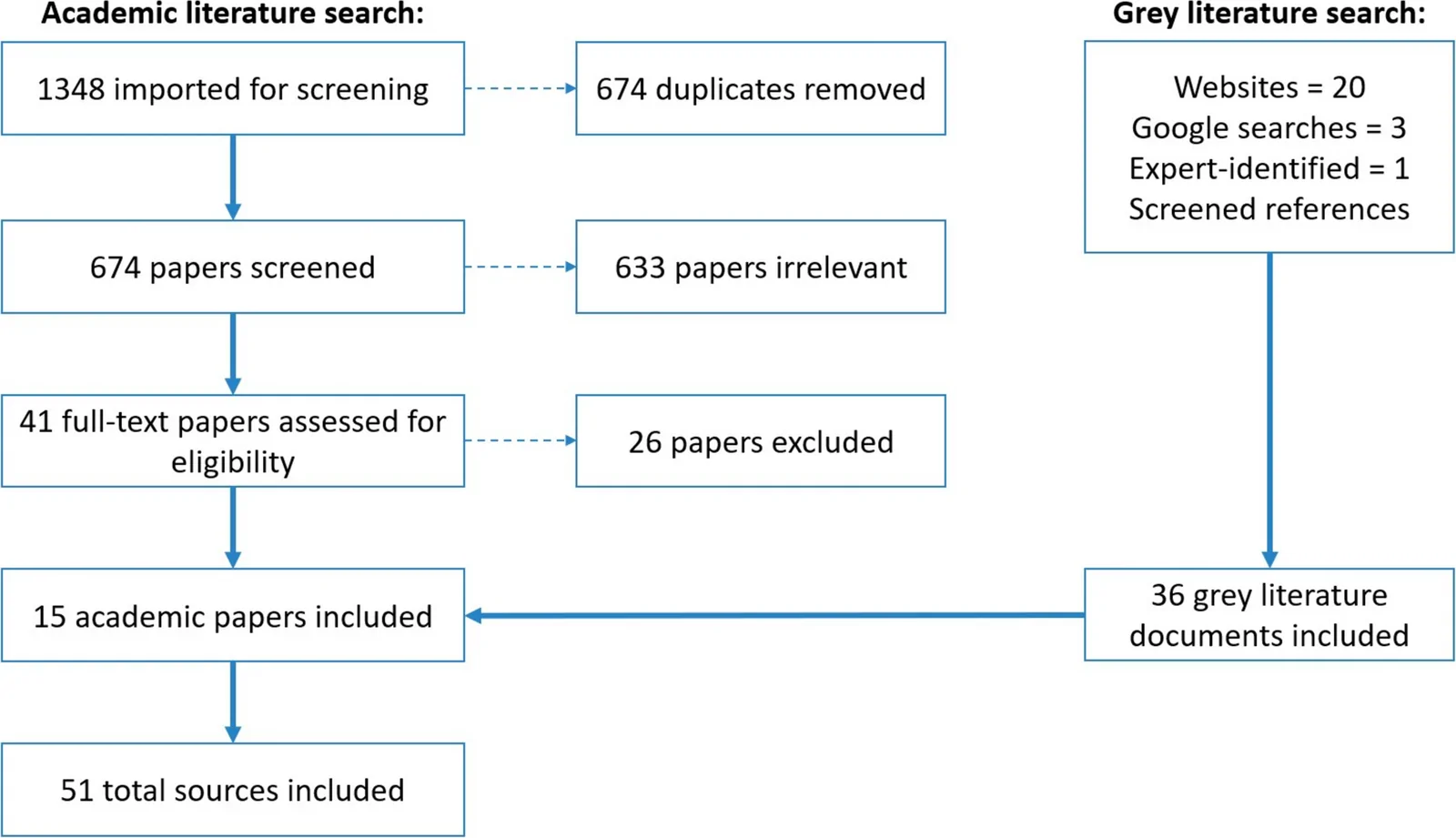
Jurisdictional review PRISMA Diagram (from Seabrook, 2024). The following PRISMA diagram demonstrates the screening process for selecting articles to include in the jurisdictional review

Snowball and respondent-driven recruitment risk overrepresentation of similar perspectives if participants refer us to individuals with similar perspectives (Patton, 2014). We minimized this risk by requesting connections to specific individuals in addition to relying on participant recommendations. We also continued recruitment until we had representation from various types of stakeholders at each level of the public health system (Appendix 1: Fig. 3).

Longitudinal policy analyses are influenced by recall bias, the risk of participants misremembering facets of the issue of interest (Walt et al., 2008). In our study, some participants could not recall whether or how the 2008 recession impacted public health budgets. Interviewing a wide range of participants from different time periods and complementing interview data with document reviews helped limit the impact of recall bias.

Finally, we were unable to assess participants’ claims about public health funding trends due to limited access to public health expenditure data in BC and the variable quality of existing sources (Ammi et al., 2021). Future work that leverages provincial accounting data (e.g., as available in Quebec (Ammi et al., 2024)) could be used to validate our qualitative findings.

## Conclusion

This study finds that despite historical budget-setting tendencies in British Columbia, public health financing is also influenced by political, structural, and external factors. Major events and crises can prompt new attention and understanding around public health with the support of strategic advocacy efforts. Outside of crisis times, we find that relationships are key to promoting the importance of public health to decision-makers, facilitated by organizational structures that include public health experts in financial decision-making. Future research could investigate other Canadian jurisdictions by testing the conceptual model developed based on the BC case, as well as comprehensively analyze the merits and feasibility of policy and practice options for promoting sustainable public health financing.

## Contributions to knowledge

What does this study add to existing knowledge?

- The main contribution of this study is its exploratory investigation of the external, structural, and political factors influencing public health financial decision-making.
- We find that external factors such as public health crises and major sociopolitical events can create windows of opportunity for investments or budget cuts.
- We also find that consolidating public health funds into budgets distinct from other health services tends to protect public health funds from reallocation.
- We heard that strong relationships between public health leaders and senior decision makers are key to increasing the priority of public health in budget-setting.

What are the key implications for public health interventions, practice, or policy?

- The findings from this study shed light on some of the possible policy strategies for sustaining public health funding, such as inclusion of public health experts in decision-making and developing public health performance indicators, which may inform current public health system strengthening efforts.

## Data Availability

Sources collected through the jurisdictional review are provided in an online Supplementary Table.

## Appendix 1

See Fig. 3.

## Appendix 2

See Table 5.

**Table 5:** Interview Guide

Section	Questions & Speaking Points
*Overview*	1. What is your current position? a. How long have you held this role, and how long have you worked in the health system/public health/BC government? 2. Can you briefly describe the process that is used to decide on the public health budget allocation? 3. Can you describe any criteria used to determine public health budgets? a. How are government/Ministry of Health priorities taken into account? b. How are changes in population needs captured? c. How is public health program effectiveness considered? d. How are health equity impacts considered?
*Stakeholder analysis*	4. What is your role in influencing decision-making for resource allocation towards public health programs? 5. Who are the most influential actors in setting annual public health budgets? 6. What is the role of public health advocates?
*Financing*	7. After the budget is set, are resources meant for public health ever redirected to other services? 8. From your perspective, how are health care priorities balanced with public health priorities? a. Who is influential in determining the balance between funding health care and public health? 9. Can you think of any reforms that have had a major impact on public health funding? (e.g. *Public Health Act,* Guiding Framework) 10. What do you think has helped maintain public health funding in BC over time? 11. Can you give an example of a time where a public health budget reduction was proposed and overcome? How was it overcome? 12. To what degree does the present allocation of funding to public health services allow medium- to long-term planning?
*Pandemic-related*	13. How were the initial allocations of crisis funding for the COVID-19 pandemic decided? a. Who were the key actors involved? 14. How has public health decision-making changed since COVID-19? Are any new factors considered? a. Are these changes temporary – will they last beyond the pandemic?
*Closing*	15. If you could adapt the current approach taken to determine the budgets for public health, what would you change? 16. Is there anything else you would like to add that we haven’t covered yet? *Thank the participant for their time. Ask participant about other possible participants for our study, and if they’re interested in validating our results*

## Appendix 3

See Fig. 4.

## Footnotes

1 The WHO outlines the horizontal functions of public health systems, based in health system building blocks, as (1) governance, (2) financing, (3) human resources, (4) health information systems, (5) research, and (6) social participation and health communication (World Health Organization, 2010).
